# Accurate diagnosis of bullous pemphigoid requires multiple health care visits

**DOI:** 10.3389/fimmu.2023.1281302

**Published:** 2023-11-27

**Authors:** Päivi Leisti, Anna Pankakoski, Jari Jokelainen, Outi Varpuluoma, Laura Huilaja, Jaana Panelius, Kaisa Tasanen

**Affiliations:** ^1^ Department of Dermatology, Research Unit of Clinical Medicine, Medical Research Center Oulu, Oulu University Hospital and University of Oulu, Oulu, Finland; ^2^ Department of Dermatology and Allergology, University of Helsinki and Helsinki University Hospital, Helsinki, Finland; ^3^ Infrastructure for Population Studies, Faculty of Medicine, University of Oulu, Oulu, Finland

**Keywords:** bullous pemphigoid, diagnosis validation, ICD-10 code, incidence, positive predictive value

## Abstract

**Introduction:**

Accurate use of diagnostic codes is crucial for epidemiological and genetic research based on electronic health record (EHR) data.

**Methods:**

This retrospective study validated the International Classification of Diseases (ICD)-10 diagnostic code L12.0 for bullous pemphigoid (BP) using EHR data from two Finnish university hospitals. We found 1225 subjects with at least one EHR entry of L12.0 between 2009 and 2019. BP diagnosis was based on clinical findings characteristic of BP and positive findings on direct immunofluorescence (DIF), BP180-NC16A enzyme-linked immunosorbent assay (ELISA) or indirect immunofluorescence (IIF) assay.

**Results:**

True BP was found in 901 patients; the positive predictive value (PPV) for L12.0 was 73.6% (95% CI 71.0-76.0). L12.0 was more accurately registered in dermatology units than any specialized health care units (p<0.001). Including patients with multiple L12.0 registrations (≥3), increased the accuracy of the L12.0 code in both dermatology units and other settings.

**Discussion:**

One diagnostic code of L12.0 is not enough to recognize BP in a large epidemiological data set; including only L12.0 registered in dermatology units and excluding cases with <3 L12.0 record entries markedly increases the PPV of BP diagnosis.

## 1 Introduction

Registry-based research is becoming more commonplace and electronic health records (EHRs) are currently widely used in medical research (1). EHRs hold a large amount of routinely collected, patient-specific data, efficient utilization of which is essential to registry-based medical research (1–3). However, EHRs are not primarily created for research and imprecise diagnoses and incorrectly entered diagnostic codes can cause errors when the data are used for secondary purposes (2, 3). Thus, it is important for EHRs used in research to maintain a high degree of validity in terms of the diagnoses of interest (3).

Bullous pemphigoid (BP) is an autoimmune blistering disease of the skin that mostly affects the elderly (4). The incidence of BP is increasing, causing a growing disease burden and rate of associated mortality (5–10). Significant risk and predisposing factors for BP and comorbidities of BP have been identified in previous registry and cohort studies (9, 11–26).

Previously, validation of BP diagnostic codes has been performed in varying settings in the UK, the United States and Sweden (3, 27, 28). Finnish health registries are considered to be reliable sources of information (29). However, field specific diagnosis validation studies are scarce, and so far, psoriasis is the only dermatological disease for which validation of diagnostic codes has been performed (30).

In the present study we validated the ICD-10 diagnostic code L12.0 for BP using EHR data obtained from two hospital districts in Finland.

## 2 Materials and methods

### 2.1 Study material

We performed a retrospective registry study based on EHRs from the Oulu University Hospital (OUH) in the Northern Ostrobothnia region of Finland and of the Helsinki University Hospital (HUH) in the Uusimaa region. Northern Ostrobothnia has an estimated population of 411 000 (31). Uusimaa is the largest and southernmost university hospital district with an estimated population of 1.7 million (31).

All consecutive patients with at least one entry of the ICD-10 diagnosis code L12.0 for BP between January 1^st^ 2009 and December 31^st^ 2019 were included in this study. We collected all available data concerning BP diagnostics, starting from the patient’s first EHR data entry until June 30^th^, 2020. Two experienced dermatology residents manually evaluated the EHRs of the cohort (AP for HUH and PL for OUH patients) and collected data on BP in a structured way based on shared predefined criteria. The EHR data included both outpatient and inpatient data in the two specialized care hospitals. Age at symptom onset, sex, and symptom duration were recorded. The data of specialty for registration of L12.0 diagnosis and the number of registrations were also collected.

**Figure 1:**
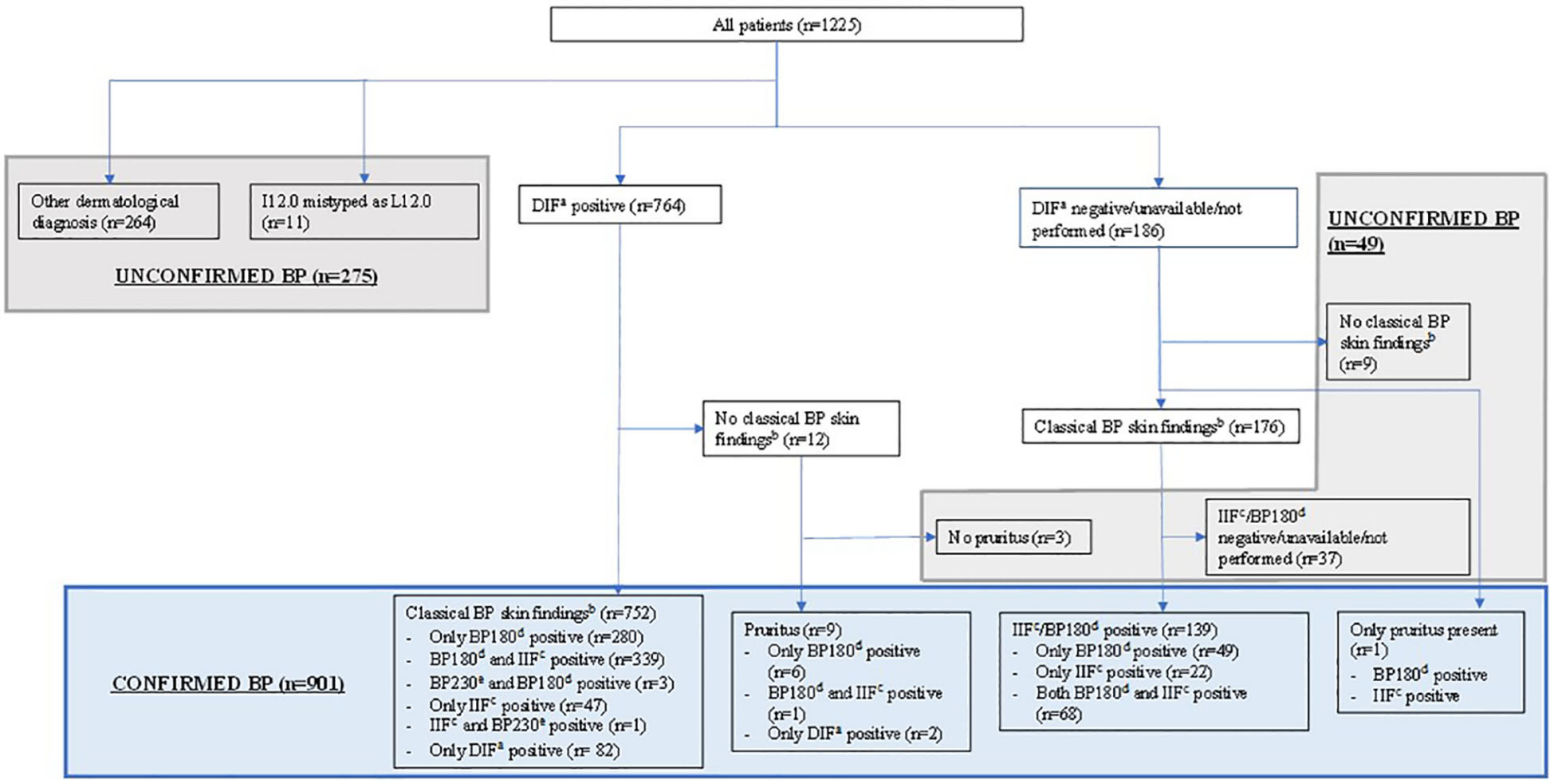
Subject disposition. At least one electronic health record entry of the International Classification of Diseases (ICD)-10 code L12.0 for BP was found for 1225 subjects in Oulu and Helsinki University Hospitals between January 1^st^ 2009, and December 31^st^ 2019. Based on characteristic cutaneous symptoms and measurable BP-compatible laboratory findings 901 of them had confirmed BP diagnosis. ^a^Linear fluorescence of immunoglobulin G (IgG) and/or complement component 3 in the basement membrane zone of a cutaneous perilesional biopsy in direct immunofluorescence. ^b^Cutaneous tense or eroded blisters or excoriations due to scratching. ^c^Circulating IgG autoantibodies against basement membrane antigens in indirect immunofluorescence ±1 month from diagnosis. Primate oesophagus as a substrate. Positive titre ≥10. ^d^Circulating BP180-NC16A antibodies in enzyme-linked immunosorbent assay. Positive value ≥9 U/ml. ^e^Circulating BP230 antibodies in indirect immunofluorescence ±1 month from diagnosis. Primate oesophagus as a substrate. Positive titre ≥10. BP, bullous pemphigoid; DIF, direct immunofluorescence; IIF, indirect immunofluorescence.

Each diagnosis of BP was confirmed based on clinical findings typical of BP (tense or eroded blisters or excoriations due to scratching) and at least one of the following criteria: 1) positive DIF; 2) positive BP180 ELISA; 3) positive IIF. These criteria were considered positive with the following findings: DIF: linear fluorescence of immunoglobulin G and/or complement component 3 in the basement membrane zone of a perilesional skin sample (Departments of Pathology, OUH and HUH). BP180 ELISA: circulating anti-BP180 NC16A domain immunoglobulin G antibodies (MBL Medical & Biological Laboratories Co., Ltd., Nagoya, Japan), value ≥9 (32). IIF: circulating immunoglobulin G autoantibodies against basement membrane antigens detected by indirect immunofluorescence ±1 month from diagnosis, with primate oesophagus as a substrate, titre ≥10 (32). Data on circulating BP230 antibody positivity were also collected, but the significance of a positive BP230 titre was only supplemental in the diagnostics, as at least one of the above-mentioned criteria was fulfilled in cases with positive BP230 (**Figure 1**). BP230 antibodies: Circulating BP230 antibodies in indirect immunofluorescence ±1 month from diagnosis, with primate oesophagus as a substrate, titre ≥10 (HUSLAB, Helsinki University Hospital/Klinisch-immunologisches Labor Prof. Dr. med. Winfried Stöcker, Germany). Patients whose only clinical manifestation was pruritus, but who had positive DIF findings were also confirmed as having BP.

### 2.2 Statistical analyses and data management

Study data were saved and managed using the REDCap electronic data capture tools hosted at the University of Oulu (33). All statistical analyses were conducted using the R (R Core Team, 2021), RStudio (Rstudio Team, 2021) and Stata (version 13.0, StataCorp. 2013. Stata Statistical Software: Release 13. College Station, TX: StataCorp LP) software packages. For details of statistical analyses, see **Supplemental Appendix S1**.

## 3 Results

### 3.1 Patients with confirmed BP diagnosis

We identified 1225 patients with at least one ICD-10 diagnostic code L12.0 for BP entered into their EHR at the OUH (n= 260) or the HUH (n= 965) (**Figure 1**). We determined that 901 patients had a correct BP diagnosis (designated as ‘BP group’) based on typical clinical and immunopathological and/or -serological findings (for details see Materials and Methods). For the remaining 324 patients BP diagnosis could not be confirmed. The mean age at diagnosis in the BP group was 76.7 years and the female to male ratio was 1.13 (**Table 1**). The age-adjusted incidence of BP in our study population was 37 cases per million per year (95% confidence interval [CI] 34-39) and the age-adjusted incidence using European standard population as a reference was 46 cases per million per year (95% CI 43-50).

**Table 1:** Demographic and clinical characteristics of patients with confirmed bullous pemphigoid.

	All cases n=901 (%)	Male n=423 (%)	Female n=478 (%)
**Age at diagnosis, mean [+/-SD]**	76.7 [11.3]	75.2 [11.6]	78.0 [10.8]
Skin symptom^a^ duration			
**<1 months**	176 (19.5)	98 (23.2)	78 (16.3)
**1-3 months**	285 (31.6)	137 (32.4)	148 (31.0)
**3-6 months**	146 (16.2)	73 (17.3)	73 (15.3)
**>6 months**	201 (22.3)	83 (19.6)	118 (24.7)
Unknown^b^	83 (9.21)	31 (7.33)	52 (10.9)
No visible skin symptoms^a^	10 (1.11)	1 (0.24)	9 (1.88)
Pruritus duration^c^			
**<1 months**	133 (14.8)	69 (16.3)	64 (13.4)
**1-3 months**	225 (25.0)	103 (24.3)	122 (25.5)
**3-6 months**	137 (15.2)	72 (17.0)	65 (13.6)
**>6 months**	211 (23.4)	84 (19.9)	127 (26.6)
Unknown^d^	124 (13.8)	55 (13.0)	69 (14.4)
**No pruritus**	71 (7.9)	40 (9.5)	31 (6.5)
Positive DIF^e^, n^f^ (%)	761 (84.5)	356 (84.2)	405 (84.7)
Positive BP180^g^, n^f^ (%)	747 (82.9)	346 (81.8)	401 (83.9)
Positive IIF^h^, n^f^ (%)	479 (53.2)	212 (50.1)	267 (55.9)
Positive BP230^i^, n^f^ (%)	4 (0.4)	1 (0.2)	3 (0.6)

SD, standard deviation; CI, confidence interval.

a Cutaneous tense or eroded blisters or excoriations due to scratching prior to diagnosis.

b Patient did not report symptom duration or symptom duration was not recorded.

c Prior to diagnosis.

d Patient did not report pruritus duration or pruritus duration was not recorded.

e Linear fluorescence of immunoglobulin G (IgG) and/or complement component 3 in the basement membrane zone of a cutaneous perilesional biopsy in direct immunofluorescence.

f Number of patients with positive test among 901 BP cases.

g Circulating BP180-NC16A antibodies in enzyme-linked immunosorbent assay. Positive value ≥9 U/ml.

h Circulating IgG autoantibodies against basement membrane antigens in indirect immunofluorescence ±1 month from diagnosis using primate oesophagus as a substrate. Positive titre ≥10.

i Circulating BP230 antibodies in indirect immunofluorescence ±1 month from diagnosis using primate oesophagus as a substrate. Positive titre ≥10.

Of the 901 patients in the BP group, 761 had findings diagnostic for BP in the DIF analysis (**Figure 1**). Nine of them presented with pruritus in the absence of any visible skin manifestations, but regardless of this, they were classified as having confirmed BP because of the positive DIF findings. In 139 patients, BP diagnoses were confirmed based on clinical symptoms (see Materials and Methods) and positive findings in IIF analysis and/or BP180 ELISA. In one patient with pruritus as the only clinical manifestation, BP diagnosis was confirmed due to positive IIF and clearly elevated levels of BP180. BP symptom duration prior to diagnosis and the number of positive diagnostic findings varied, see **Table 1**.

### 3.2 Patients with unconfirmed or incorrect BP diagnosis

Of all cases featuring a BP diagnosis code, 324 patients did not have BP. In 264 cases, another dermatological diagnosis was found instead of BP, 102 of whom had another autoimmune blistering dermatosis (**Figure 1**, **Table 2**; **Supplemental Table SI**). In 11 patients, the BP diagnostic code L12.0 was present, but had most likely been entered in error, with the intention of entering the code I12.0, as there was no evidence of BP or any other dermatological disease, but all 11 records contained other entries of the code I12.0.

**Table 2:** Other dermatological diagnoses which were found in 264 of the 324 patients without confirmed bullous pemphigoid.

	All, n	Male, n (%)	Female, n (%)
**Patients with other cutaneous diagnosis** **^a^**	264	95 (36.0)	169 (64.0)
**Mucous membrane pemphigoid**	61	13 (21.3)	48 (78.7)
**Infective or nummular eczema**	19	8 (42.1)	11 (57.9)
**Pemphigus**	13	5 (38.5)	8 (61.5)
**Linear IgA dermatosis**	11	4 (36.4)	7 (63.6)
**Eczema staticum cruris**	9	5 (55.6)	4 (44.4)
**Atopic eczema**	7	4 (57.1)	3 (42.9)
**Prurigo nodularis**	5	0 (0.0)	5 (100.0)
**Urticaria**	4	1 (25.0)	3 (75.0)
**Scabies**	2	2 (100.0)	0 (0.0)
**Other blistering dermatoses** **^b^**	77	32 (41.6)	45 (58.4)
**Other or undefined dermatitis** ^c^	51	19 (37.3)	32 (62.7)
**Undefined pruritus**	12	3 (25.0)	9 (75.0)

a Overall 271 diagnoses; 1 patient had 3 different diagnoses, and 5 patients had 2 different diagnoses.

b Included other autoimmune blistering diseases; epidermolysis bullosa acquisita (n=4), dermatitis herpetiformis (n=8) and pemphigoid gestationis (n=5).

c For specific diagnosis distribution see **Supplemental Table SI**.

### 3.3 Positive predictive value of BP diagnosis

The positive predictive value (PPV) for the ICD-10 diagnostic code for BP, L12.0, in the whole study population was 73.6% (95% CI 71.0%-76.0%). The optimal cut-off value for accurately identifying BP was the presence of ≥3 registrations of L12.0 in a patient’s record. At this point the PPV was 85.0 (95% CI 83.0%-87.0%), the sensitivity 85.1% (95% CI 83.1%-87.1%) and the specificity 58.3 (95% CI 55.6%-61.1%).

**Figure 2:**
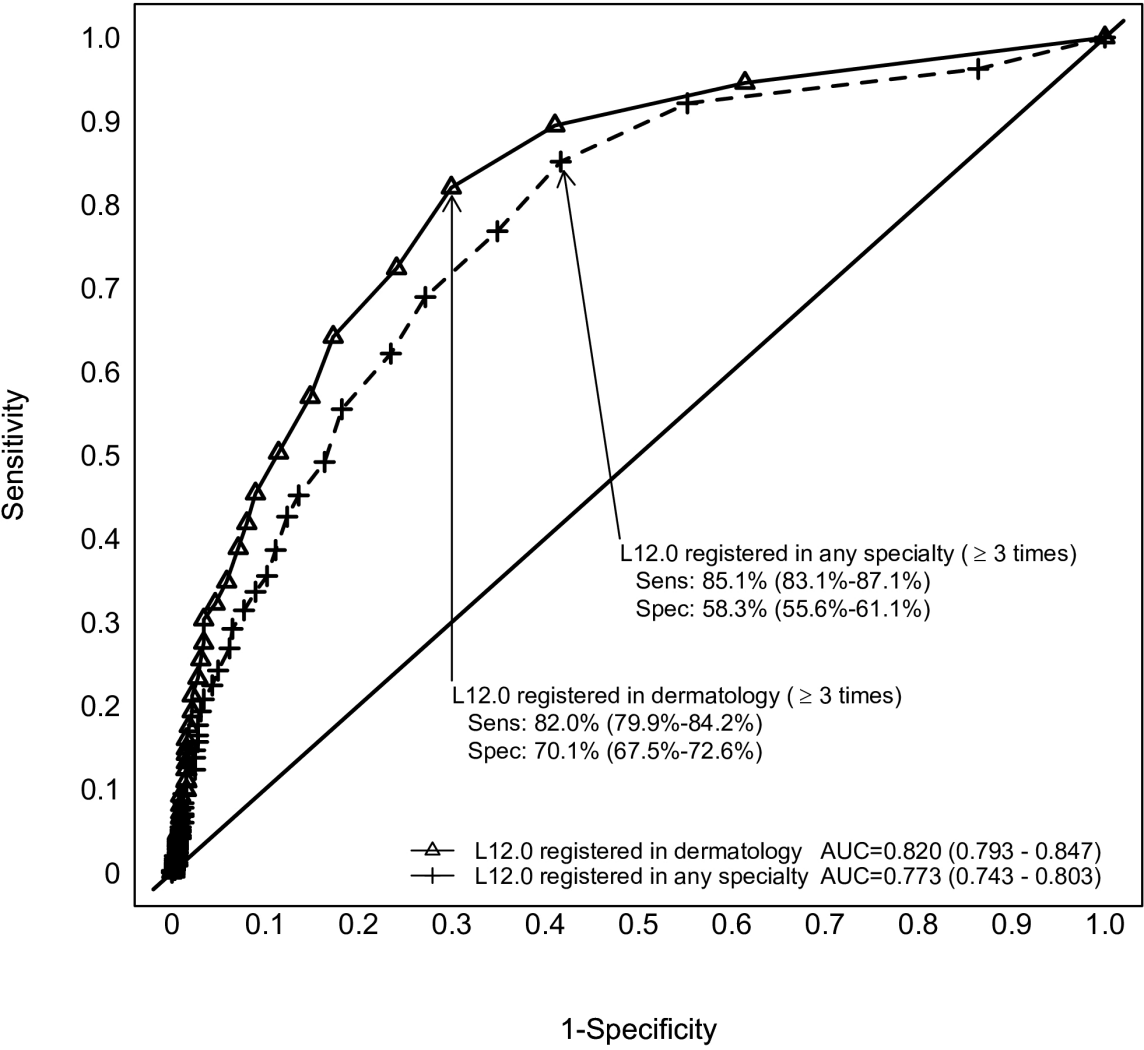
Multiple visits increase the accuracy of bullous pemphigoid diagnosis. Reciever operating characteristic curve for the accuracy of L12.0 diagnoses registered in a dermatology department and those registered in any specialized healthcare setting. 95% Confidence Intervals in brackets. Difference between AUCs, p<0.001. AUC, Area under curve; Sens, Sensitivity; Spec, Specificity.

The analysis of the specialty setting of the registration of L12.0 found that diagnoses recorded in dermatology units were significantly more accurate than those registered in any specialized health care unit. The area under curve was 0.82 (95% CI 0.79-0.85) for registrations made in dermatology units, and 0.77 (95% CI 0.74-0.80) for those made in any setting (p<0.001; **Figure 2**). Again, for diagnoses registered in dermatology units, the optimal cut-off number of L12.0 registrations in a patient’s record was ≥3, with the PPV being 88.4% (95% CI 86.6%-90.2%), the sensitivity 82.0% (95%CI 79.9%-84.2%), and the specificity 70.1% (95% CI 67.5%-72.6%).

## 4 Discussion

In our study, the PPV for L12.0 in the whole study population was 73.6% (95% CI 71.0%-76.0%). The validity was determined based on strict diagnostic criteria, including characteristic cutaneous symptoms and measurable BP-compatible laboratory findings. In a further analysis of specialty of registration, we discovered that L12.0 codes registered at dermatology units were significantly more accurate than those registered in any specialized health care setting. The optimal cut-off point for determining BP in a dataset like ours, was the presence of at least three entries of L12.0 for registrations made at either dermatology units, or any specialized health care setting.

To the best of our knowledge, BP diagnostic codes have previously been validated in only three studies (3, 27, 28), only one of which validated ICD-10 diagnostic codes for BP (28). This study validated L12.0, L12.8 and L12.9 ICD-10 codes for 307 BP patients retrieved from the Swedish National Patient Register (specialized inpatient and outpatient care). This study found PPV values for the codes as high as 92% (28). However, our study’s diagnostic criteria for BP were more stringent than those of the Swedish study: we did not consider positive histopathological findings alone sufficient to diagnose BP and thus we excluded patients without other diagnostic findings from the BP group. Furthermore, in the Swedish study, 21 patients were diagnosed as having true BP based solely on a dermatologist’s clinical evaluation of the patients’ records. Moreover, 16 patients were excluded from the PPV analysis because their medical records were missing and could not be evaluated. Had the Swedish study included these patients with missing data in the analysis as ‘unconfirmed’ cases (as per our own methodology), the PPV would have been 87.6%.

The study from the UK validated the primary health care diagnostic codes of BP using linked secondary healthcare inpatient data and ICD-10 codes for BP as the diagnostic benchmark (3). This study found the PPV for BP diagnostic Read codes in general practice to be 93.2% (95% CI 91.3-94.8) (3). This study is not comparable to ours since it did not validate the ICD-10 codes for BP in the secondary healthcare setting. In the US study, the ICD-9 codes 694.5 for BP and 694.4 for pemphigus were validated in a large specialized in- and outpatient dataset (27). This study found that only 28 (29%) of 97 patients with a single entry of the code 694.5 had clinical or diagnostic findings that supported the BP diagnosis. In our study, the PPV for L12.0 was higher in the whole study population. As did we, *Hsu et al.* found that one entry of the BP diagnostic code was not sufficient accurately to recognize BP in a large epidemiological data set. The PPV of multiple entries of the 694.5 code in the study by *Hsu et al.* was 99% (95% CI 93%-99%), but the number of diagnostic codes registered was not specified (27). However, this study cannot be directly compared to ours, both because it validated an ICD-9 code (rather than an ICD-10 code) and because, unlike our study, it accepted typical histopathological findings alone to confirm a BP diagnosis.

There are several reasons why a quarter of patients in our study population did not have true BP, the main one being that the code L12.0 was recorded in some cases when BP was initially suspected, but another dermatological diagnosis was eventually later confirmed (**Table 2**). In other cases, clerical errors led to the L12.0 code being used mistakenly. In a few cases, the diagnoses were recorded in an institution not covered by our study methodology, and we did not have access to diagnostic data from the beginning of a patient’s disease course, which made BP verification per our study methodology impossible. These patients were therefore assigned to the non-BP group. It is also possible that in some cases, the patients’ symptoms were atypical of BP, or were not recorded in their EHR. Per our methods, these patients were necessarily excluded from the BP group based on the lack of cutaneous signs matching our criteria. Lastly, some cases were excluded from our BP group because the required diagnostic examinations had not been performed for justifiable reasons.

The significantly greater reliability of L12.0 registered in dermatology units compared with those from any other specialty is not surprising, since dermatologists are familiar with BP, its diagnostic pathways and correct coding of the suspected disease. Diagnostic tests are also usually performed in dermatology departments. However, the reason why the area under the curves do not differ more greatly, is because L12.0 codes registered in other specialties are usually copied from those set by dermatologists.

Since routinely collected data is ever more frequently used for research purposes, it is important, that when documenting data on patient records, clinicians should use a definite diagnostic code only once the diagnosis is confirmed, not when a case is only suspected. Furthermore, accuracy needs to be maintained in the use of codes when diagnosing different types of pemphigoids.

The incidence of BP has varied from 2.1 to 42.8 per million per year internationally and is increasing (5, 34–37). In more recent studies, the incidence in the UK and in Sweden was 76.3 and 71 per million per year, respectively (9, 38), and in a systematic review and meta-analysis of 27 studies, the pooled annual cumulative incidence of BP was 8.2 cases per million people in 23 studies and the pooled incidence rate was 34.2 million per year in four studies globally (39). We can also confirm the rising trend in BP incidence since the incidence in our current study population was 46 cases per million per year, 2.6 times higher than the age-adjusted incidence of 18 cases per million per year between 1987 and 2013 that we found in our previous study (25). There are several possible explanations for the increasing overall incidence of BP, including an ageing population, increased use of certain drugs that carry a risk for BP (especially dipeptidyl peptidase-4inhibitors, or gliptins, used to treat type 2 diabetes), increasing prevalence of comorbid conditions of BP, improvements in diagnostic tools, and increasing awareness of the different clinical variants of BP (4, 5).

A major strength of this study is its large study population. We were able to analyze all patient records manually and data were collected in a structured and systematic way based on predefined criteria. In addition, the two regions included are geographically distant and inhabited by distinct population bases. We were also able to precisely define the number of registered diagnostic codes and the specialty of registration and to analyze the impact of these on the PPV.

Including patients treated in specialized care units only can lead to selection bias. However, as the diagnosis of BP typically requires diagnostic tests (e.g., DIF) not available in primary healthcare units in Finland, we do not consider this to be a major limitation of our study. In addition, the large study population and long follow-up period may compensate for this. Furthermore, while the accuracy of data on symptoms varies with the individual documenting clinician, the immunological, histopathological, and serological data required by our study are unambiguous.

We conclude that one recorded entry of diagnostic code of L12.0 is not enough to recognize BP when querying a large epidemiological data set. Including only L12.0 registered in dermatology units and limiting the set to cases with at least three registrations of L12.0, markedly increases the positive predictive value of the L12.0 code. However, if the data on registration specialty are not available, it is also reasonable to include patients with at least three entries of L12.0 regardless of where the code was recorded. It will benefit epidemiological future studies of BP if the above-mentioned matters are considered in the planning.

## Data availability statement

The data analyzed in this study is subject to the following licenses/restrictions: All data used in this article are based on patients’ personal electronic health records, and by Finnish legislation are thus classified as sensitive information. Thus, datasets related to this article cannot be shared. On case-to-case basis, based on application, the local authorities may grant access to data (www.findata.fi/en). Requests to access these datasets should be directed to www.findata.fi/en.

## Author contributions

PL: Conceptualization, Funding acquisition, Resources, Writing – review & editing, Formal Analysis, Investigation, Methodology, Visualization, Writing – original draft. AP: Investigation, Resources, Visualization, Writing – original draft, Writing – review & editing, Funding acquisition. JJ: Writing – original draft, Writing – review & editing, Data curation, Formal Analysis, Software, Visualization. OV: Writing – review & editing, Supervision, Investigation, Writing – original draft. LH: Supervision, Writing – review & editing, Conceptualization, Visualization. JP: Investigation, Supervision, Writing – review & editing. KT: Writing – review & editing, Conceptualization, Funding acquisition, Project administration, Resources, Supervision, Visualization.
