# Supplementary material for: Accurate diagnosis of bullous pemphigoid requires multiple health care visits
Source: Front Immunol. 2023 Nov 27;14:1281302. doi: 10.3389/fimmu.2023.1281302 (PMC10711056; doi:10.3389/fimmu.2023.1281302)
Supplement: Supplementary file 1 [file DataSheet_1.pdf]

Supplementary Material

Accurate Diagnosis of Bullous Pemphigoid Requires Multiple Health Care Visits

Päivi Leisti MD, Anna Pankakoski MD, Jari Jokelainen MSc, Outi Varpuluoma MD, PhD, Laura Huilaja MD, PhD, Jaana Panelius MD, PhD, and Kaisa Tasanen MD, PhD*.

*** Correspondece:** kaisa.tasanen@oulu.fi

1 Supplementary Data

Appendix S1

Statistical analyses and data management

Baseline characteristics are stratified by sex and presented as frequencies (%) for categorical data and means and standard deviations for continuous data. Age-specific incidence was calculated, and standardized incidence was calculated by applying direct age standardization to the 2013 European standard population (1), using 5-year age bands up to 90 years of age.

We calculated the sensitivity, specificity, and positive predictive value with 95% CI for the number of registered L12.0 diagnoses (2). To measure the efficacy of the number of L12.0 diagnoses and to select an optimal threshold value (cut-off point), we calculated Youden's index and generated a receiver operating characteristics (ROC) curve and area under the ROC curve (3,4). P values <0.05 were considered to be statistically significant.

References

1. Pace M, Lanzieri G, Glickman M, Grande E, Zupanic T, Wojtyniak B, et al. Eurostat Revision of the European Standard Population. Report of Eurostat's task force. 2013. [Internet]. 2013 [cited 2017 Sep 13]. Available from: http://ec.europa.eu/eurostat/documents/3859598/5926869/KS-RA-13-028-EN.PDF/e713fa79-1add-44e8-b23d-5e8fa09b3f8f

2. Shapiro DE. The interpretation of diagnostic tests. Stat Methods Med Res 1999;8(2):113–134.

3. Fluss R, Faraggi D, Reiser B. Estimation of the Youden Index and its Associated Cutoff Point. Biom J 2005;47(4):458–472.

4. Youden WJ. Index for rating diagnostic tests. Cancer 1950;3:32–35.

2 Supplementary Figures and Tables

2.1 Supplementary Tables

Table SI. Diagnosis distribution of patients without confirmed bullous pemphigoid

**Other blistering dermatoses, n:**	**77**
Blistering conditions of oral cavity (suspected mucous membrane pemphigoid [diagnosis not confirmed], lichenoid oral mucositis, lichen planus of oral cavity, aphtous stomatitis and unspecified blistering of oral cavity)	20
Unspecified blistering of skin	15
Dermatitis herpetiformis	8
Lichen planus	5
Pemphigoid gestationis	5
Epidermolysis bullosa acquisita	4
Porfyria cutanea tarda	3
Edema blisters	3
Bullosis diabeticorum	2
Phytophotodermatitis	2
Vasculitis	2
Pyoderma gangrenosum	1
Erythema fixum	1
Insect bite reaction	1
Granulomatous cheilitis	1
Cutaneous drug reaction	1
Pyoderma	1
Herpes Zoster	1
Erythema multiforme	1
**Other or undefined dermatitis, n:**	**51**
Undefined dermatitis	30
Eczema	4
Skin infection	3
Acne	1
Skin cancers and solar keratoses (multiple diagnoses in one patient)	1
Seborrhoeic dermatitis susp	1
Ulcerative oral infection	1
Subacute Cutaneous Lupus Erythematosus	1
Lymphomatiod papulosis	1
Perioral dermatitis/rosacea	1
Granulomatous infection	1
Lichen planopilaris	1
Acute generalized exanthematous pustulosis	1
Psoriasis	1
Vasculitis	1
Pompholyx and contact dermatitis	1
Infiltratio lymphocytica atypica pro obs mycosis fungoides	1
